# Exon shuffling and alternative splicing of ROCO genes in brown algae enables a diverse repertoire of candidate immune receptors

**DOI:** 10.3389/fpls.2024.1445022

**Published:** 2024-08-23

**Authors:** Linhong Teng, Yuhuan Sun, Jiayi Chen, Chenghui Wang, Jonathan M. Urbach, Bostjan Kobe, Naihao Ye, Qiangcheng Zeng

**Affiliations:** ^1^ College of Life Sciences, Dezhou University, Dezhou, China; ^2^ Ragon Institute of Mass General Brigham, MIT, and Harvard, Cambridge, MA, United States; ^3^ School of Chemistry and Molecular Biosciences, Institute for Molecular Bioscience and Australian Infectious Diseases Research Centre, University of Queensland, Brisbane, QLD, Australia; ^4^ National Key Laboratory of Mariculture Biobreeding and Sustainable Production, Yellow Sea Fisheries Research Institute, Chinese Academy of Fishery Sciences, Qingdao, China

**Keywords:** brown algae, ROCO gene family, immunity, phylogenetics, exon shuffling

## Abstract

The ROCO family is a family of GTPases characterized by a central ROC-COR tandem domain. Interest in the structure and function of ROCO proteins has increased with the identification of their important roles in human disease. Nevertheless, the functions of most ROCO proteins are still unknown. In the present study, we characterized the structure, evolution, and expression of ROCOs in four species of brown algae. Brown algae have a larger number of ROCO proteins than other organisms reported to date. Phylogenetic analyses showed that ROCOs have an ancient origin, likely originated in prokaryotes. ROCOs in brown algae clustered into four groups and showed no strong relationship with red algae or green algae. Brown algal ROCOs retain the ancestral LRR-ROC-COR domain arrangement, which is found in prokaryotes, plants and some basal metazoans. Remarkably, individual LRR motifs in ROCO genes are each encoded by separate exons and exhibit intense exon shuffling and diversifying selection. Furthermore, the tandem LRR exons exhibit alternative splicing to generate multiple transcripts. Both exon shuffling and alternative splicing of LRR repeats may be important mechanisms for generating diverse ligand-binding specificities as immune receptors. Besides their potential immune role, expression analysis shows that many ROCO genes are responsive to other stress conditions, suggesting they could participate in multiple signal pathways, not limited to the immune response. Our results substantially enhance our understanding of the structure and function of this mysterious gene family.

## Introduction

The ROCO protein family was originally described in 2003 in *Dictyostelium discoideum* (Bosgraaf and Van Haastert, 2003). Subsequently, ROCO proteins were identified in a wide range of organisms from prokaryotes to eukaryotes. All the ROCO proteins possess a Ras-of-complex (ROC) domain and a C-terminal-of-Roc (COR) dimerization domain. The ROC domain belongs to the class of small G-proteins, with high sequence similarity to Ras, although phylogenetic analysis shows that ROC domains are clearly distinct from other Ras-like GTPases (Bosgraaf and Van Haastert, 2003). Apart from typical ROC-COR, most ROCOs contain a kinase domain and a diverse set of regulatory and protein-protein interaction domains. Leucine-rich repeats (LRRs) are present in most ROCO proteins. LRRs were generally described as domains that mediate protein interactions (Kobe and Kajava, 2001), suggesting they may interact with partners of the ROCO proteins (Marín et al., 2008). In addition, ankyrin, WD40, and other types of repeats are also often present in the N-terminal region of ROCO proteins, and may also be involved in protein-protein interactions. The presence of several interaction domains within ROCO protein sequences may reduce the requirement for separate adaptor proteins in pathways involving ROCO proteins (Tomkins 2018).

Research on ROCO proteins significantly intensified since the identification of links between ROCO proteins and human disease, notably, leucine-rich repeat kinase 2 (LRRK2) with Parkinson’s disease (PD) and death-associated protein kinase 1 (DAPK1) with cancer (Marín, 2006; Terheyden, 2018). Extensive research was then started to investigate the structure and functions of other ROCO proteins. During past years, two comprehensive reviews on ROCO proteins were published (Civiero et al., 2014; Wauters et al., 2019). However, most of the published studies citing them focused on the LRRK2 protein, whereas most ROCO proteins have not been investigated yet.

The origin of ROC domains within prokaryotes is uncertain, but eukaryotic ROCOs have been suggested to have a symbiotic, mitochondrial origin (Marín, 2006). Their presence in a wide range of species, both archaea and several distant bacterial groups suggests a more ancient origin predating the mitochondrial endosymbiosis. Prokaryotic ROCO proteins possess N-terminal LRRs and a C-terminal ROC-COR unit (Deyaert et al., 2019). The LRR-ROC-COR multidomain arrangement is a broadly distributed domain architecture, and is found in prokaryotes, plants and some metazoans. Archaea also possess ROC domains with a simple LRR-ROC-COR architecture (Marín et al., 2008), suggesting this domain combination is very ancient and crucial to the function of ROCO proteins.

ROCO proteins have multiple putative functions. The role in intracellular signaling was proposed based on the presence of both GTPase and kinase domains (Marín et al., 2008). In *D. discoideum*, 11 ROCO genes were identified and among them the functions of GbpC, Pats1 and QkgA have been studied in detail. They are involved in multiple cellular processes, including chemotaxis, cell division, and development (Abysalh et al., 2003; Kortholt et al., 2012). Only a small number of ROCOs have been detected in vertebrates, including LRRK1, LRRK2, DAPK1, and MFHAS1 in humans. LRRK2 has been implicated in a diverse range of cellular processes, including cytoskeletal dynamics and macroautophagy. Mutations in LRRK2 are associated with familial PD or other neurodegenerative diseases (Marín, 2006; Cookson, 2016). LRRK1, a close paralog of LRRK2, has been associated with many distinct cellular mechanisms. Mutations in LRRK1 are less detrimental than in LRRK2 (Marín, 2008). DAPK1 is linked to cell death pathways and functions as a tumor suppressor (Inbal et al., 1997). Plants contain one or two ROCO genes (Bosgraaf and Van Haastert, 2003), but their functions are poorly understood. One ROCO gene, TRN1 from *Arabidopsis thaliana*, has been studied in detail, and its mutants possess altered growth and morphogenesis phenotypes (Cnops et al., 2006). Although ROCO proteins have aroused growing interest, studies have largely focused on the human disease-related genes, such as LRRK2 and DAPK1. Published studies represent the tip of the iceberg with regards to the roles of ROCO proteins and much more remains to be uncovered regarding other ROCO proteins.

Multicellular brown algae belong to the SAR supergroup (Stramenopiles, Alveolates, and Rhizarians), which originated from secondary endosymbiosis events (Keeling, 2010). Brown algae are the biggest photoautotrophic marine organisms and constitute the major primary producers in coastal ecosystems (Thomas et al., 2014). Kelps, such as *Saccharina* and *Macrocystis*, play an increasingly important role in the aquaculture industry (Zhang et al., 2021b; Teng et al., 2023). ROCO proteins of brown algae were firstly identified in *Ectocarpus* (Zambounis et al., 2012); they consist of N-terminal LRRs followed by a ROC-COR domain. The authors found that the LRRs of ROCOs exhibit a repetitive intron-exon structure and suggested that *Ectocarpus* ROCO proteins may be involved in immunity. Brown algae, together with other SAR species, diverged from plants and animals about one billion years ago (Cock et al., 2012). Gene transfer from endosymbionts to the host has built a complex genomic mosaic in the SAR supergroup (Dorrell et al., 2017). In the present study, we explore the origin of brown algal ROCO proteins and their evolutionary relationships with ROCO proteins of other phyla. The availability of additional brown algal genomes and transcriptomes facilitates an exhaustive survey of ROCO genes, and provides new insights into the functional mechanisms of ROCO genes. The analysis provides a detailed picture of the ROCO gene family in brown algae and further provides a reference for studying brown algal immunity.

## Materials and methods

### Identification of ROCO genes in brown algae

The genomes of four brown algae (*Ectocarpus*, *Saccharina japonica*, *Cladosiphon okamuranus*, and *Nemacystus decipiens*) were retrieved from public databases. Genome sequences and RNA transcript data including splicing variants of *Ectocarpus* version V2016 were downloaded from the website http://bioinformatics.psb.ugent.be/orcae/overview/Ectsi (Cormier et al., 2016). Genomes of *C. okamuranus* and *N. decipiens* were downloaded from http://marinegenomics.oist.jp/algae/ (Nishitsuji et al., 2016, Nishitsuji et al., 2019). The genome for *S. japonica* was downloaded from https://bioinformatics.psb.ugent.be/. The LRR domain profile PF00560 was downloaded from the Pfam website. The HMMER3 software (Mistry et al., 2013) was used to search for LRR domains in the proteome of each brown alga using the PF00560 as a query. The acquired sequences were searched for ROCO proteins using the ROC profile PF08477 as a query. The candidate ROCO proteins were submitted to the online InterProScan program to further confirm the domain composition.

### Phylogenetic analysis

Due to the extensive domain shuffling and recombination of the repetitive LRR motifs of ROCO proteins, phylogenies based on alignment of full-length ROCO proteins proved uninterpretable. Therefore, we constructed the phylogenetic trees using the extracted ROC domains. Firstly, we constructed the phylogenetic tree of brown algal ROCOs to explore their classification. The ROC domains of the four brown algae were extracted, then aligned using MUSCLE V5 (Edgar, 2021). The ML tree was constructed using RAxML-NG with the JTT+G4 model predicted by ModelTest-NG (Kozlov et al., 2019). Bootstrapping with 1000 resamplings was performed to obtain the confidence support value. To trace the origin of brown algal ROCO proteins in a broader context, the phylogenetic tree including more organisms was constructed. The organisms we used to search the ROCO proteins reached almost all phyla in the tree of life, including green algae, red algae, plants, metazoan, SAR organisms, prokaryotes and protists. Their genomes were downloaded from JGI or NCBI. The ROCO proteins from these species were searched using the ROC profile PF08477. The resulting proteins were manually checked using the InterProScan to exclude non-ROC proteins. And then the ROC domains were extracted. Together with brown algal ROC domains, the big phylogenetic tree was constructed using the same procedure used in the brown algal-only ROC tree building.

### Sequence analysis

The domain composition of ROCO proteins was identified using InterProScan online. Notably, the COR domain (PF16095) of brown algae was not identified by InterProScan, so we performed the hmmsearch to identify the COR domain using PF16095 as a query. Intron and exon information of ROCO genes was extracted from GFF files of the four brown algae. For each ROCO protein, subcellular localization was predicted using Euk-mPLoc 2.0 http://www.csbio.sjtu.edu.cn/bioinf/euk-multi-2/ (Chou and Shen, 2010). Molecular weights and isoelectric points were calculated using the ProtParam tool https://web.expasy.org/protparam/. The protein transmembrane helices were predicted by DeepTMHMM https://services.healthtech.dtu.dk/service.php?DeepTMHMM. Sequence logos for LRR motifs were generated using TBTOOLS (Chen et al., 2020). The alternative splicing variants of *Ectocarpus* were acquired from genome sequences version V2016 and were displayed using the genome browser tool Artemis (Carver et al., 2012). A 3D model of the ROCO protein SJ02233 was generated using the online AlphaFold2 https://neurosnap.ai/service/AlphaFold2.

### Expression of ROCO genes in *Ectocarpus* and *S. japonica*

The expression patterns of ROCO genes under different life-cycle stages and various abiotic stresses were examined using the available transcriptome data of *Ectocarpus* and *S. japonica*. The RNA-seq data of haploid gametophytes and diploid sporophytes were used to compare the expression of genes between different life stages (Lipinska et al., 2019). Furthermore, previous microarray data of the *Ectocarpus* transcriptome (Dittami et al., 2009; Ritter et al., 2014) were used to explore the expression changes of ROCO genes in response to abiotic stresses, including copper stress, hyposaline stress, hypersaline stress, and oxidative stress. The stress responses of ROCO in *S. japonica* under high light, high temperature, acidification, hyposaline and hypersaline conditions were explored using digital gene expression (DGE) library sequencing (Zhang et al., 2021a). Genes with a P-value < 0.05 and a log2 (fold change) >1 were considered as significantly differentially expressed genes. Hierarchical cluster heatmaps were created using the R package.

## Results

### Identification of ROCO genes and phylogenetic analysis

A total of 111 ROCO genes was identified in four brown algae, including 31 genes in *Ectocarpus*, nine genes in *S. japonica*, 22 genes in *C. okamuranus*, and 47 genes in *N. decipiens* (**Supplementary Table S1**). All the genes have an N-terminal LRR domain and a C-terminal ROC domain. The average length of the ROCO proteins is 1241 amino acids (aa). The length of ROC domain ranges from 77 to 467 aa, with the average length of 192 aa. The large range of ROC domain lengths is a result of truncations or insertions within the ROC domain. For example, in the long ROC domain of Cok_S_s158_12713.t1, non-conserved ROC sequences are inserted in the conserved ROC domain. The number of tandem LRR motifs ranges from 4 to 49. Notably, the COR domain was not detected in these genes by the online InterProScan. According to a hmmsearch of the COR profile PF16095, 18 out of the 111 genes possess the conserved COR domain. The length of COR domain ranges from 112 to 189 aa, with the average length of 150 aa. Gene structure analysis shows that brown algal ROCO genes have multiple exons, ranging from 7 to 55 exons, with the average number of 20 exons. ROCO genes are also found in other SAR organisms, albeit in smaller numbers in each species.

**Figure 1:**
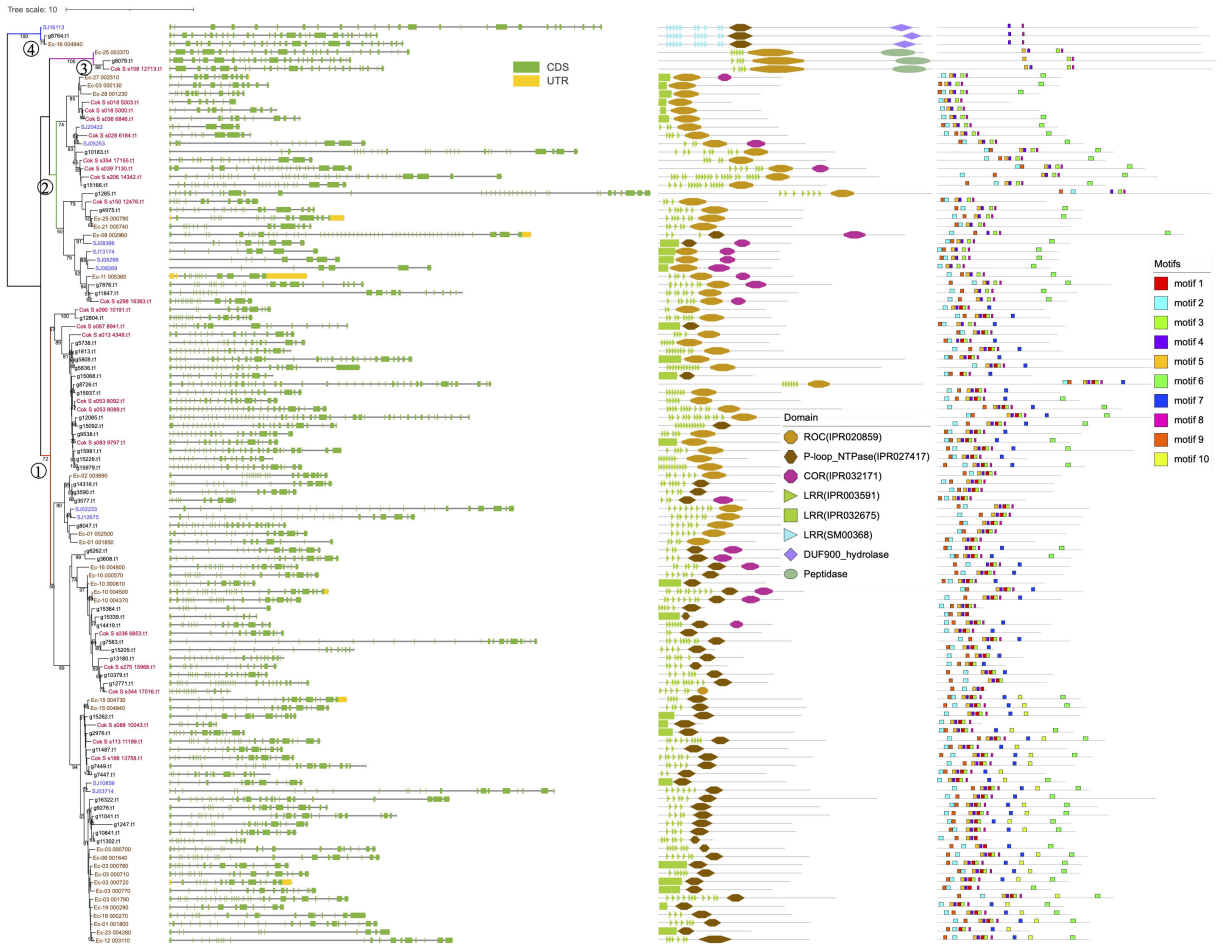
Phylogenetic tree of the 111 brown algal ROC domain sequences. The ML tree was generated using RaxML-NG with the JTT+G4 model predicted by ModelTest-NG. Numbers on the nodes represent the bootstrap values larger than 50%. The four ROCO groups are numbered as 1-4. Exon-intron structures, domain architecture and conserved motifs are shown next to the tree.

The 111 brown algal genes clustered into four groups (**Figure 1**). Groups 1 and 2 contain most of the ROCO members, while group 3 contains three members, with an extra peptidase domain(IPR009003) at the C-terminus. Group 4 also contains three members and possesses a different subfamily of LRRs (SM00368) from those of groups 1 and 2 (IPR003591) and a C-terminal DUF900 domain (IPR010297).

**Figure 2:**
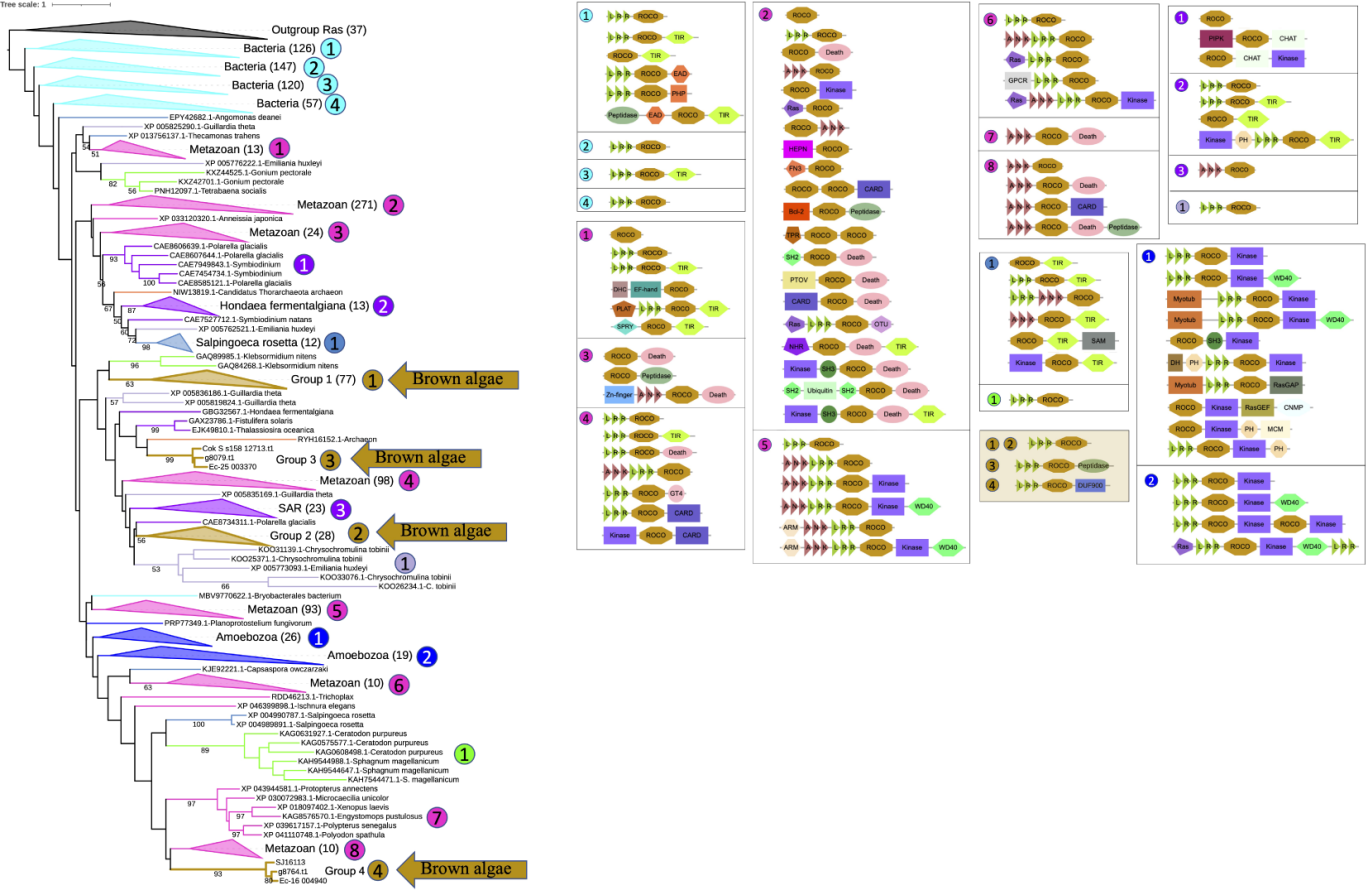
Rooted phylogenetic tree and domain analysis of ROCO sequences across a wide range of kingdoms. A total of 1231 ROC domain sequences and 37 Ras sequences were aligned and the ML tree was generated using RAxML-NG with the LG+G4 model predicted by ModelTest-NG. The domain combination in each clade are shown beside the tree. Branch colors represent different kingdoms of life.

To trace the origin of brown algal ROCO proteins in a broader context, phylogenetic trees including more organisms were constructed. Firstly, we generated a hidden Markov model using the ROC domains of brown algal ROCOs. And we downloaded 464,830,651 protein sequences in the NR database of NCBI. Then we performed an hmmsearch using the HMM profile of brown algal ROC domains with the sequence reporting threshold of 1e-15. A total of 13,085 sequences were obtained. Considering the ROC profile may also detect other GTPases, such as Ras and Rho, we manually checked their domain composition using InterProScan, and deleted the non-ROC sequences. Then 11,268 ROCO proteins were obtained. They were clustered using CD-hit with an identity threshold of 0.6, resulting in 2821 sequences. The ROC domains of these sequences were extracted and clustered again using the CD-hit with an identity threshold of 0.6. The resulting 1120 sequences, together with brown algal ROC domains, were aligned and the rooted ML tree was constructed, with the brown algal Ras domain as outgroup. In this rooted tree using more representative sequences in NR database, most ROCO proteins are from prokaryotes and metazoans. The four groups of brown algal ROCOs are distributed in separated branches. Notably, ROCOs from bacteria are in the basal position, suggesting that ROCOs originated from prokaryotes. Domain analysis shows that LRR-ROCO are the prevalent domain architecture in prokaryotes. Brown algal ROCOs keep this typical and ancient LRR-ROCO structure, though they do not group closely with prokaryotic ROCOs on the tree. More diverse domain architectures are present in animals, in which as many as forty domain combinations can be identified (**Figure 2**).

To determine whether brown algal ROCOs are derived from secondary endosymbiosis of green or red algae, or alternatively are shared with their nearest SAR cousins and therefore most likely inherited from SAR ancestors, we used ROC domains from other SAR species, green algae, red algae, *Dictyostelium*, and representative sequences of top hits from BLASTP against the NR database to build the phylogenetic tree, with the Ras sequences of brown algae as an outgroup (**Supplementary Figure S1**). We used ROC profile PF08477 as a query to do hmmsearch in SAR species, green algae and red algae. ROCOs exist in several SAR species, such as diatoms, oomycetes, and *Schizochytrium aggregatum*, with no more than four copies in each species. Searches in green algae and red algae reveal that only multicellular algae possess ROCO genes. These proteins possess relatively simple domain architectures compared to the complex domain combinations in *Dictyostelium* ROCOs and animal LRRK or DAPK, e.g., LRR-ROCO (*Chara braunii* and *Klebsormidium nitens*), ANK-ROCO (*Gonium pectoral*), or TPR-ROCO-TIR (*Chondrus crispus*). To find the ROC genes most closely related to the ones in brown algae, we performed several rounds of online BLASTP on the NCBI website using representative brown algal ROC domains as the queries (**Supplementary Table S2**). The top 100 protein hits were primarily from bacteria, animals and *Pythium*. They were downloaded from NCBI and clustered using CD-hit with an identity threshold of 0.9; the resulting 42 sequences were combined with the sequences of SAR species, green algae and red algae for phylogeny reconstruction. The 267 sequences of ROC domains, together with 23 Ras domain sequences as an outgroup, were aligned and the ML tree was generated. In the tree topology, sequences are generally clustered by the species classification. As in the brown algal-only ROCO tree presented in **Figure 1** and big tree in **Figure 2**, the four ROCO groups of brown algae are still separate and distinct from each other, and showed no strong relationship with green, red algae or other SAR species, with the exception of groups 3 and 4. Members of group 3 have peptidase domains in their C-termini and were nested within a clade including ROCOs from other SAR organisms, some of which also have C-terminal peptidase domains. The three members of group 4 clustered with one sequence from a diatom, and they all have C-terminal DUF900 domain, suggesting these two types of ROCOs may have been inherited from a common ancestral SAR species, although somewhat divergence occurred in brown algae. Notably, ROCOs from bacteria are still in the basal position, further supporting a presumed origin of ROCOs in prokaryotes. The phylogeny of the COR domain of ROCO sequences exhibits similar status with the phylogeny of the ROC domain. COR sequences from the same organisms cluster together, and the CORs between group 1 and group 2 are still separated (**Supplementary Figure S2**). The tree topology suggests that the four groups of ROCOs exist in ancestral SAR organisms, and were then lost in some lineages. Domain analysis on the tree shows that LRR-ROCO is the prevalent, most widely distributed domain architecture of ROCO proteins, especially in brown algae and prokaryotes. N-terminal ANK repeats are found in oomycetes and the green alga *Gonium*. TPR domain are found in *Chondrus*. N-terminal kinase, death, TIR, and helicase domains are found in different organisms. By contrast, brown algae exhibit the relatively simple domain combination of LRR-ROCO.

From the tree topology and domain analysis, we can see that ROCO proteins are an ancient family, which may have originated from the common ancestor of prokaryotes and eukaryotes. The central LRR-ROCO domain combination in these proteins is likely ancient and maintained but expanded in brown algae, while other diverse domains may have been acquired independently in each organism.

To see if other domain combination exists in ROCOs of brown algae, we again used the HMM profile of brown algal ROC domains as a query to perform hmmsearch, this time with the default E value of 10.0 and obtained 491 target sequences. The phylogenetic tree generated from these sequences includes subfamilies ROC, Ras, ARF/Rab, Rho/TIF/OBG of the small GTPase superfamily and ATPase superfamilies (**Supplementary Figure S3**). The clade of the ROC family stands out as a separate group among the superfamily of small GTPases, clearly distinguished from the other four families. Furthermore, no other ROCO domain combination was found, suggesting that LRR-ROC-COR is the only domain structure in brown algal ROCOs.

### Exon shuffling of LRR motifs

**Figure 3:**
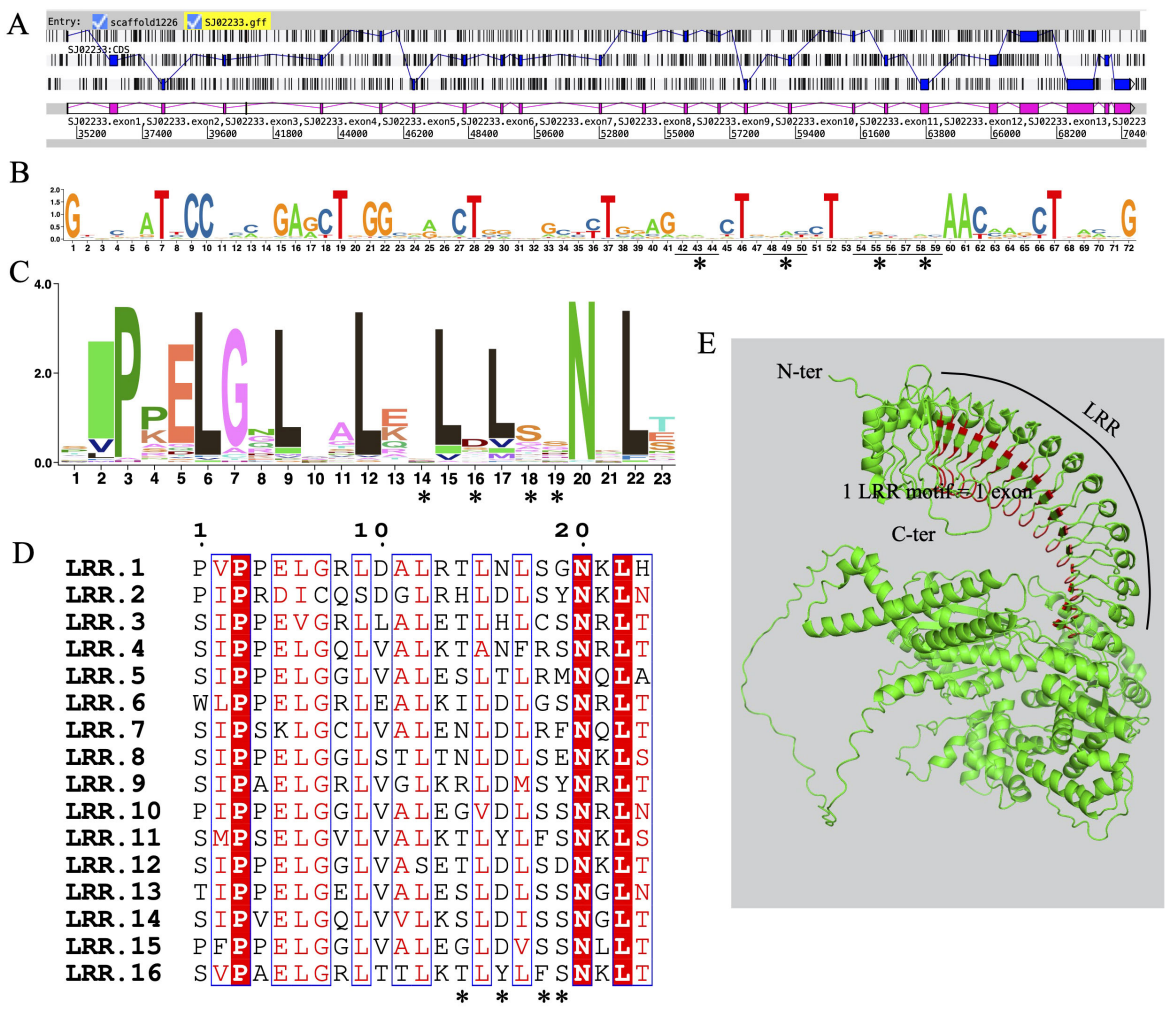
Sequences and structures of LRR domains from ROCO genes in S. japonica. **(A)** Genomic organization of SJ02233 reveals intense shuffling of LRR-encoding exons. **(B)** Consensus sequence logos of a total of 62 LRR exons, each containing 72 bp nucleotides. **(C)** Consensus sequence logos of a total of 62 LRR motifs, each containing 23 amino acids encoded by the nucleotides 3-71 of each exon. **(D)** Alignment of the 16 exons composing the LRR domain of SJ02233, showing the conserved residues interspersed with variable amino acids. Asterisks represent the four amino acids positions subject to positive selection revealed by the M2 and M8 models. **(E)** 3D model of SJ02233 predicted using AlphaFold2. Sites shown in red are the four positively selected sites. The sites are located on the concave face of the β-sheet, the probable ligand-binding face of the domain.

**Figure 4:**
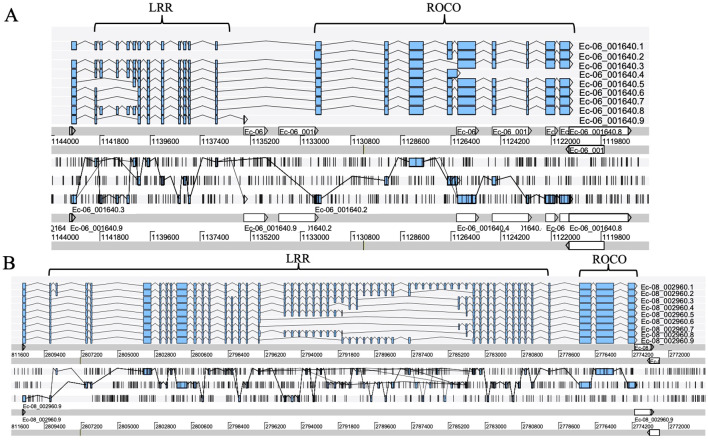
Alternative splicing products of the ROCO genes Ec-06_001640 **(A)** and Ec-08_002960 **(B)**, each with different LRR domain contents. Sequence data were obtained from the reannotation results of the *Ectocarpus* genome reported by Cormier et al. (2016). Exons are represented as filled boxes, introns as lines. Different splicing events are represented as lines connecting exons.

Exon shuffling data was previously reported in *Ectocarpus* (Zambounis et al., 2012). Here, we revisit the gene structure of the four brown algae. The ROCO genes (except for groups 3 and 4) exhibit strong exon shuffling. Each exon contains 72 bp and is in phase 2. One exon ranging from nucleotides 3 to 71 encodes a LRR of 23 amino acids, which contains a conserved 17-residue segment with the consensus sequences LxxLxxLxxLxLxxNxL(x can be any amino acid and L positions can also be replaced by valine, isoleucine and alanine) (**Figures 3A–D**). The alternative splicing data shows that the genes with shuffling LRR exons have multiple splicing variants, for example Ec-06_001640 and Ec-08_002960 of *Ectocarpus* (**Figure 4**). These variants have diverse combinations of LRR motifs, which could generate diverse ligand-binding specificities. In order to confirm the functional significance of exon shuffling, we performed online 3D modeling of the ROCO protein SJ02233 from *S. japonica*. The LRR domain of SJ02233 is predicted to adopt a repetitive parallel β-sheet structure, each repeat consisting of a β-strand and an α-helix connected by loops. The parallel structure forms a curved arc or horseshoe-shaped molecule with the β-sheet lining the inner concave face (**Figure 3E**). We also tested for the diversifying selection acting on the shuffling LRR domains using the site model (M1 vs. M2, M7 vs. M8) in PAML (**Supplementary Table S3**). Four sites (14, 16, 18, 19) were predicted to be under positive selection, which is consistent with the result of ROCO in *Ectocarpus* (Zambounis et al., 2012). The four positively selected sites are located on the concave side of LRR repeats, suggesting that these sites could be directly related to the evolution of new ligand-binding specificities.

### Expression analysis of ROCO genes

**Figure 5:**
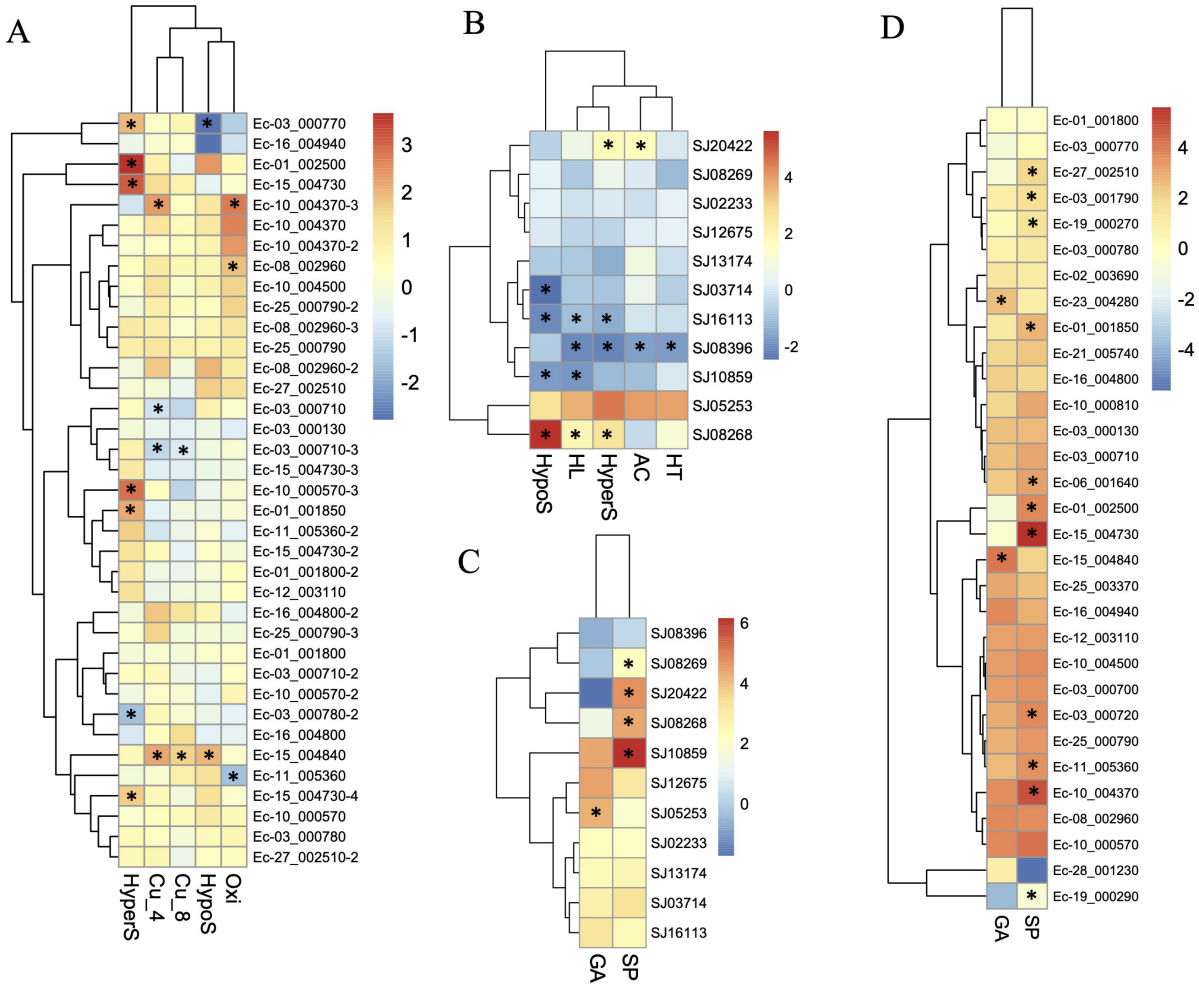
Expression profiles of ROCO genes in *Ectocarpus* **(A, D)** and *S. japonica* **(B, C)**. **(A, B)** Log2-transfromed fold changes of the expression levels compared to the control. **(C, D)** Log10-transfromed TPM (transcripts per million) values. Black star indicates the significantly differently expressed genes compared to the control or the other life stage (fold change > 2, p <0.05, t-test).

To further understand the functional roles of ROCO proteins in brown algae, we analyzed the gene expression profiles of the ROCO genes in the two brown algae *Ectocarpus* and *S. japonica* (**Table 1**; **Figure 5**). Nineteen ROCO genes in *Ectocarpus* were present in the microarray data, and 11 of them had two to four contigs/singletons. Hierarchical clustering revealed that several ROCO genes are responsive to stress conditions. Six genes were significantly upregulated, while one gene was downregulated under hypersaline stress (fold change >2 and p-value < 0.05). One gene was upregulated, while one gene was downregulated by hyposaline stress. Notably, one gene (Ec-01_002500) was upregulated by both hypersaline and hyposaline stress, and one gene (Ec-03_000770) was significantly upregulated by hypersaline while downregulated by hyposaline stresses, suggesting the genes may participate in the salt signaling pathway. Two genes were upregulated, while one gene was downregulated by oxidative stress. Two genes were upregulated and two genes were downregulated under copper stress. Among them, one gene (Ec-15_004840) was upregulated by copper stress of both 4 hours and 8 hours, and one gene (Ec-03_000710) was downregulated by copper stress of both 4 hours and 8 hours. From the patterns above, we infer that at least two genes (Ec-01_002500 and Ec-03_000770) may have potential roles in the salt response pathway and two genes (Ec-15_004840 and Ec-03_000710) may participate in the copper response pathway. Hierarchical clustering of *S. japonica* ROCO genes showed that two genes were upregulated and four genes were downregulated by stress conditions. Notably, SJ08268 and SJ08396 were influenced by multiple stress conditions. SJ08268 was upregulated under low salt, high salt and high light stresses, while SJ08396 was downregulated under high light, high salt, acidification, and high temperature conditions, suggesting the two genes could play crucial roles in stress-responsive networks. However, responses to stressors can lead to global changes in gene expression, so a gene whose expression responds to a particular stressor may be not actually a meaningful player in the response or they are downstream of the relevant response pathway. More genetic methods are needed to elucidate their roles in these stressors. Divergent expression levels were also observed in different life stages. In *Ectocarpus*, eleven genes were highly expressed in sporophytes (SP), while two genes were highly expressed in gametophytes (GA). For *S. japonica*, four genes were sporophytes-biased while one gene was gametophytes-biased. Collectively, more genes are highly expressed in sporophytes compared to gametophytes.

Table 1The detailed expression values and statistics of differentially expressed ROCO genes in *Ectocarpus* and *S. japonica*.

**Table:** 

Gene ID	TPM (GA)	TPM (SP)	log2FoldChange (SP/GA)	pvalue	padj
Ec-01_001850	2.16	6.51	1.94	0.00	0.00
Ec-11_005360	6.00	11.17	1.28	0.00	0.00
Ec-01_002500	0.60	13.25	4.79	0.00	0.00
Ec-10_004370	12.63	28.93	1.59	0.00	0.00
Ec-19_000270	1.24	2.90	1.58	0.00	0.00
Ec-15_004840	17.51	4.28	-1.60	0.00	0.00
Ec-15_004730	0.77	48.30	6.32	0.00	0.00
Ec-06_001640	4.84	9.03	1.28	0.00	0.00
Ec-03_001790	1.89	3.34	1.19	0.00	0.00
Ec-03_000720	7.91	13.08	1.11	0.00	0.00
Ec-23_004280	5.80	1.95	-1.14	0.01	0.01
Ec-19_000290	0.07	0.63	2.94	0.00	0.00
Ec-27_002510	0.62	3.95	2.96	0.00	0.00
SJ05253	18.57	3.82	-1.87	0.00	0.00
SJ20422	0.30	26.30	7.13	0.00	0.00
SJ08268	2.61	21.23	3.44	0.00	0.00
SJ10859	20.92	71.46	1.94	0.00	0.00
SJ08269	0.93	4.86	2.87	0.00	0.00

Data from Lipinska et al., 2019. TPM: transcripts per kilobase of exon model per million mapped reads

**Table:** 

Gene ID	Condition	RPKM	log2FoldChange	pval	padj
SJ08268	Con	15.17359			
	HypoS	437.4219	5.629176	4.19E-19	7.47E-17
	HL	53.51954	1.95766	0.000785	0.009012
	HyperS	95.1102	2.658753	3.15E-06	0.000216
SJ10859	Con	892.388			
	HypoS	158.1475	-1.71391	0.000159	0.00121
	HL	231.039	-1.8184	3.77E-05	0.000791
SJ08396	Con	139.0346			
	AC	50.23922	-1.63848	0.002744	0.048485
	HL	32.91407	-2.00546	0.000257	0.003781
	HT	46.53296	-1.72623	0.001502	0.023933
	HyperS	32.08796	-2.13269	0.000132	0.004186
SJ16113	Con	247.9107			
	HypoS	36.70475	-1.99801	1.25E-06	1.82E-05
	HL	107.737	-1.0885	0.003346	0.027213
	HyperS	94.44194	-1.40548	0.000321	0.008227
SJ03714	Con	227.1891			
	HypoS	24.63375	-2.47049	1.31E-06	1.90E-05

Data from Zhang et al. 2021a. RPKM: reads per kilobase of exon model per million mapped reads.

**Table:** 

Gene ID	Condition	Normalized expression	log2FoldChange	pval
Ec-15_004730	Con	43.39584		
	HyperS	368.2413	3.08	0.028
Ec-15_004730-4	Con	636.6641		
	HyperS	2087.261	1.71	0.008
Ec-11_005360	Con	508.7174		
	Oxi	171.5529	-1.56	0.018
Ec-15_004840	Con	154.372		
	HypoS	621.1525	2.008	0.033
	Con_4	343.7432		
	Cu_4	1616.576	2.232	0.01
	Con_8	706.7696		
	Cu_8	2219.009	1.65	0.049
Ec-03_000780-2	Con	9542.071		
	HyperS	3186.97	-1.586	0.002
Ec-01_001850	Con	3216.841		
	HyperS	14330.86	2.155	0.001
Ec-10_000570-3	Con	415.3863		
	HyperS	2984.452	2.844	0.002
	Con_8	1771.102		
	Cu_8	756.998	-1.227	0.28
Ec-03_000710	Con_4	1459.545		
	Cu_4	744.6286	-0.971	0.006
Ec-08_002960	Con	355.4299		
	Oxi	1341.698	1.916	0.04
Ec-10_004370-3	Con	531.1694		
	Oxi	3495.323	2.718	0.04
	Con_4	711.6216		
	Cu_4	3580.153	2.33	0.01
Ec-01_002500	Con	31.63884		
	HyperS	399.6878	3.658	0.027
Ec-03_000770	Con	324.2043		
	HyperS	1237.802	1.929	0.017
	HypoS	46.87266	-2.795	0.013

Data from Dittami et al., 2009 and Ritter et al., 2014.

## Discussion

### Brown algae possess diverse ROCO gene repertoires with an LRR-ROC-COR domain architecture

Brown algal species contain a greater number of ROCO genes than other species. Only a few ROCO proteins have been found in vertebrates. In humans, only four ROCO proteins have been identified (LRRK1, LRRK2, DAPK1, and MFHAS1), whereas 11 ROCO proteins are present in *D. discoideum*. We have identified ten ROCOs in the multicellular green algae *Chara braunii*, and nineteen genes in *Gonium pectoral*. For red algae, only five ROCOs are found in the multicellular red alga *Chondrus crispus*. Whereas the brown algal species surveyed in this work contain between 9 and 47 ROCO genes, their unicellular relatives among SAR species possess four or fewer ROCOs. The diversity of ROCOs in multicellular algae relative to their unicellular relatives roughly parallels the phylogenetic distribution of brown algal NB-ARC genes (Teng et al., 2023), and suggests that brown algal ROCOs may participate in functions related to multicellularity such as programmed cell death in the context of multicellular innate immunity.

Genome sequence and annotation quality can affect gene identification. 37 ROCOs were reported in *Ectocarpus* in a previous study (Zambounis et al., 2012). There are actually 35 genes in total, because two pseudogenes Esi0027_0052 and Esi0102_0087 are fragments of Esi0027_0029 and Esi0036_0149, respectively. In the new version of the *Ectocarpus* genome, they correspond to Ec-03_000780 and Ec-27_002510, respectively. Some other genes, such as Esi0562_0010 and Esi0112_0048, have no conserved N-terminal LRR domain or ROC domain, so they were not included among the ROCO genes of the new annotated genome data. Compared to the other three brown algae, more ROCO genes were identified in *N. decipiens*. One possibility is that the fragmented assembled genomes may result in high gene numbers, because individual genes may be split into multiple genes during annotation. The average length of ROCO proteins in *N. decipiens* is 1269 aa, similar to *Ectocarpus* (1243 aa), *S. japonica* (1251 aa) and *C. okamuranus* (1203 aa). We also found the apparently larger number of immunity-related NB-ARC genes in *N. decipiens* (Teng et al., 2023). Unlike the tandem duplication mechanism of gene expansion that occurred in NB-ARC genes, most ROCO genes are dispersed in different scaffolds, suggesting that segmental duplication may be responsible for the increase of ROCOs in *N. decipiens*.

A larger number of ROCO genes in an organism may mean that they are involved in more pathways or may facilitate more diversity in response to diverse pathogens. Another possibility is that there are more pseudogenes. The rapid evolution of disease resistance genes may result in a high proportion of pseudogenes (Meyers et al., 2005). Twenty of the 37 ROCOs in *Ectocarpus* were previously predicted to be putative pseudogenes (Zambounis et al., 2012). The refined new version genome predicts ten ROCO pseudogenes. According to the expression levels of RNA-sequencing data, three presumed pseudogenes, Ec-01_001800, Ec-28_001230, and Ec-03_000770, have low expression levels of below 2 TPM in both life stages. In the microarray data of *Ectocarpus*, 19 of the 31 genes were present, suggesting low or no expression of the pseudogenes. For *S. japonica*, one gene SJ05253 shows almost no expression in five stress conditions of sporophytes, whereas it is upregulated in gametophytes, suggesting that rather than being a pseudogene, it is expressed only under particular conditions. However, many identified brown algal ROCO genes may be pseudogenes, particularly where they are most numerous, as they are in *N. decipiens*.

Despite the high number of ROCO genes in brown algae, the sequence composition is relatively simple, compared to the complex domain architecture in other species. ROCO proteins were classified into three groups based on their domain composition (Bosgraaf and Van Haastert, 2003; Wauters et al., 2019). Brown algal ROCOs belong to the first group, which shows the simplest domain arrangement, with the ROCO domain preceded by an N-terminal LRR domain. To date, the most diverse domain architectures have been observed in the slime mold *Dictyostelium* and the placozoan *Trichoplax adhaerens* (Civiero et al., 2014). In *Dictyostelium* ROCOs, the COR domain is succeeded by a kinase domain and the ROC domain is preceded by 3-16 LRRs. They are surrounded by other diverse domains, such as DEP, WD40, RhoGEF, and PH (Bosgraaf and Van Haastert, 2003). In the ancient placozoan *T. adhaerens*, at least 17 ROCO genes have been identified; the ROC-COR domains are surrounded by diverse functional domains, including LRRs, TPRs, CARD, and death domains. Despite the diverse domain combinations found in these organisms, the LRR-ROC-COR domains are central to the action of nearly all ROCO proteins (Deyaert et al., 2019). In our phylogenetic analysis, the domain architecture showed no strong correlation with the phylogeny. N-terminal LRRs and a C-terminal ROC-COR unit architecture seems to be the most simple and typical structure; it was identified in brown algae, some SAR species, prokaryotes, some metazoans, and green algae, suggesting that brown algae, like prokaryotes and basal plants, keep the simple and ancient ROCOs structure, while other organisms have developed more complex domain compositions during evolution. For example, the similar kinase domains of ROCOs in *D. discoideum* and LRRK2 in vertebrates were suggested to be acquired independently in a process of convergent evolution (Marín, 2008). The slime molds and placozoa were also predicted to have acquired diverse ROCO genes independently (Civiero et al., 2014).

### Exon shuffling and alternative splicing of LRRs may contribute to the generation of ROCO protein functional diversity

Although ROCO proteins have attracted considerable interest, their biological functions are still poorly understood. The expression patterns of ROCO genes in the present study suggested their involvement in the response to various stress conditions. Extensively studied ROCO genes in *D. discoideum* revealed their involvement in chemotaxis and control of cytoskeleton dynamics (van Egmond et al., 2008; Lewis, 2009). Roles of ROCOs in immune response mechanisms were reported for human ROCO proteins. For example, LRRK2 and MASL1 were shown to be upregulated on pathogen infection (Gardet et al., 2010; Ng et al., 2011), though the molecular mechanisms to modulate inflammatory response are still unclear. The LRRs of LRRK2 and MASL1 were suggested to function as cytoplasmic receptors in response to various danger signals (Hakimi et al., 2011). Zambounis et al. reported that the intense exon shuffling of LRRs underpins the variability of LRR domain in ROCO genes, and brown algae may generate their immune repertoire via somatic recombination (Zambounis et al., 2012). We further confirmed this exon shuffling structure in all the four brown algae. The striking arrangement gives us a hint that ROCOs in brown algae may be involved in immune response mechanisms. Consistent to this point, three ROCO genes were found to be upregulated in the kelp *Macrocystis pyrifera* by the treatment of 1-octen-3-ol, a kind of oxylipin which was found to induce defense reactions in plants (Zhang et al., 2021b).

LRR motifs generally comprise 20-29 residues and are present in a number of proteins with an astonishing variety of functions, including proteins involved in signal transduction, DNA repair and cell adhesion, extracellular matrix proteins, and transmembrane receptors (Andrade et al., 2001; Kobe and Kajava, 2001). LRRs are thought to be involved in protein-protein interactions, by forming non-globular structures with a parallel β-sheet lining the inner concave surface, which provides an ideal structural framework required for molecular interactions (Kobe and Deisenhofer, 1994). Most LRR domains consist of 2-45 leucine-rich repeats (Ng et al., 2011). In the current study, ROCO proteins have about ten LRRs on average, with as many as 49 tandem LRRs found in *Ectocarpus* (Ec-08_002960). Many LRR containing proteins are associated with innate immunity in plants, invertebrates and vertebrates. The LRR domains of plant NB-LRR (nucleotide-binding-LRR) type disease resistance proteins (R [resistance] proteins) are involved in specific recognition of host protein modifications mediated by pathogen effector molecules. The β-sheet portion of the LRR domain is often the ligand-binding interface and under diversifying selection in many plant NB-LRR proteins (DeYoung and Innes, 2006). In animals, Toll-like receptors (TLRs) and NOD-like receptors (NLRs), through their LRR domains, sense molecular determinants from a diverse set of bacterial, fungal and parasite components. In humans, at least 34 LRR proteins are involved in diseases (Ng et al., 2011). Repeat domains such as LRR domains have been suggested to offer evolutionary advantages over non-repeat domains (Andrade et al., 2001). The repetitive structure of LRR should be beneficial for the rapid generation of new variants required because it can evolve more rapidly when facing diverse pathogens (Kobe and Kajava, 2001). More importantly, intragenic tandem duplication through exon shuffling enables new variants to develop rapidly new binding specificities, without sacrificing old ones. A large number of repeats may reflect the avidity and cooperativity of substrate binding (D'Andrea and Regan, 2003).

We infer that exon shuffling of LRRs may provide a mechanism to evolve diverse binding specificities rapidly, while maintaining a stable β/α arc structure. Consistent with this idea, as many as nine alternative splicing transcripts are presented in ROCO genes of *Ectocarpus*; in these, the assembly of multiple LRR exons forms different combinations. Alternative splicing occurs widely in eukaryotes and can provide the main source of transcriptome and proteome diversity in an organism (Yang et al., 2016). Production of proteins with diverse domain rearrangements from the same genes represents the major alternative splicing mechanism for pathogen-resistance genes (Mastrangelo et al., 2012). Molecular analysis of transcripts encoding animal TLRs and plant R genes reveals many cases of alternative splicing, which represents a crucial aspect of signaling (Jordan et al., 2002). Unprecedented expansion of alternative splicing has been employed by arthropods to generate diverse DSCAM (down syndrome cell adhesion molecule) receptors. Several duplications of exons generated three large tandem arrays of Ig domain exons that are alternatively spliced, allowing for expression of tens of thousands of DSCAM isoforms (Schmucker and Chen, 2009). The alternative splicing of DSCAM in the mosquito immune system changes in response to various immune challenges (Smith et al., 2011). We further searched the shuffling LRR exons in the whole brown algal genomes, based on the 72 bp exon length. Sequences having shuffling LRR exons exist in their genomes, for example, 15 sequences with shuffling LRR exons (excluding ROCO genes) were found in *S. japonica*. Most of them contain only LRR motifs, which can be a reservoir for LRR shuffling. It has been suggested that exon shuffling of LRRs in ROCOs and TPRs in NB-TPR genes in *Ectocarpus* could be a hallmark of somatic recombination and form the basis of an adaptive immune system in brown algae (Zambounis et al., 2012). However, as illustrated for NB-TPR genes (Teng et al., 2023), somatic recombinant ROCO gene loci have not yet been reported in brown algae, nor have site-specific recombinases analogous to those of the vertebrate VDJ recombination system, nor specialized proliferative clonal recombinant immune cells that could support an enduring immune memory. Based on the observation of splice variants of ROCO genes in *Ectocarpus*, a simpler explanation would be that combinatorial use of alternatively spliced LRR domains enables ROCOs of brown algae to generate more binding specificities, which resembles the case in NB-TPR genes (Teng et al., 2023). Interestingly, the shuffling of LRR exons also exist in animals. Similar sized exons in LRR domains of rat luteinizing hormone receptor genes suggested that the LRR domain evolved by exon duplication and shuffling from a single prototypic exon corresponding to one LRR (Koo et al., 1991; Kobe and Deisenhofer, 1994). Notably, all of the introns of the gonadotrophin receptors are in-phase, being phase 2, the same as the phase 2 exon-intron structure of the LRRs in brown algal ROCOs, suggesting that brown algae and vertebrates share the same exon shuffling mechanisms in LRR evolution.

## Conclusions

In conclusion, we comprehensively analyzed the phylogeny and structure of the ROCO proteins in brown algae. The results show that ROCO proteins in brown algae have an ancient origin and simple domain combination, but have more proteins compared to other species. Exon shuffling and alternative splicing of the LRR motifs could potentially expand the ligand-binding specificities. However, the true nature of these genes is not yet understood, nor the role of their shuffling exons, and will require more study.

## Data Availability

The original contributions presented in the study are included in the article/**Supplementary Material**. Further inquiries can be directed to the corresponding author.

## References

[B1] Abysalh J. C. Kuchnicki L. L. Larochelle D. A. (2003). The identification of pats1, a novel gene locus required for cytokinesis in Dictyostelium discoideum. Mol. Biol. Cell 14, 14–25. doi: 10.1091/mbc.e02-06-0335 12529423 PMC140224

[B2] Andrade M. A. Perez-Iratxeta C. Ponting C. P. (2001). Protein repeats: structures, functions, and evolution. J. Struct. Biol. 134, 117–131. doi: 10.1006/jsbi.2001.4392 11551174

[B3] Bosgraaf L. Van Haastert P. J. (2003). Roc, a Ras/GTPase domain in complex proteins. Biochim. Biophys. Acta (BBA)-Molecular Cell Res. 1643, 5–10. doi: 10.1016/j.bbamcr.2003.08.008 14654223

[B4] Carver T. Harris S. R. Berriman M. Parkhill J. McQuillan J. A. (2012). Artemis: an integrated platform for visualization and analysis of high-throughput sequence-based experimental data. Bioinformatics 28, 464–469. doi: 10.1093/bioinformatics/btr703 22199388 PMC3278759

[B5] Chen C. Chen H. Zhang Y. Thomas H. R. Frank M. H. He Y.. (2020). TBtools: an integrative toolkit developed for interactive analyses of big biological data. Mol. Plant 13, 1194–1202. doi: 10.1016/j.molp.2020.06.009 32585190

[B6] Chou K. C. Shen H. B. (2010). Cell-PLoc 2.0: an improved package of web-servers for predicting subcellular localization of proteins in various organisms. Natural Sci. 2, 1090–1103. doi: 10.4236/ns.2010.210136 18274516

[B7] Civiero L. Dihanich S. Lewis P. A. Greggio E. (2014). Genetic, structural, and molecular insights into the function of ras of complex proteins domains. Chem. Biol. 21, 809–818. doi: 10.1016/j.chembiol.2014.05.010 24981771 PMC4104024

[B8] Cnops G. Neyt P. Raes J. Petrarulo M. Nelissen H. Malenica N.. (2006). The TORNADO1 and TORNADO2 genes function in several patterning processes during early leaf development in Arabidopsis thaliana. Plant Cell 18, 852–866. doi: 10.1105/tpc.105.040568 16531491 PMC1425859

[B9] Cock J. M. Sterck L. Ahmed S. Allen A. Amoutzias G. Anthouard V.. (2012). Chapter 5: The Ectocarpus genome and brown algal genomics (San Diego, CA: Elsevier Academic Press).

[B10] Cookson M. R. (2016). Structure, function, and leucine-rich repeat kinase 2: On the importance of reproducibility in understanding Parkinson’s disease. Proc. Natl. Acad. Sci. 113, 8346–8348. doi: 10.1073/pnas.1609311113 27422551 PMC4968709

[B11] Cormier A. Avia K. Sterck L. Derrien T. Wucher V. Andres G.. (2016). Re-annotation, improved large-scale assembly and establishment of a catalogue of noncoding loci for the genome of the model brown alga Ectocarpus. New Phytol. 214, 219–232. doi: doi:10.1111/nph.14321 10.1111/nph.14321 27870061

[B12] D'Andrea L. D. Regan L. (2003). TPR proteins: the versatile helix. Trends Biochem. Sci. 28, 655–662. doi: 10.1016/j.tibs.2003.10.007 14659697

[B13] Deyaert E. Leemans M. Singh R. K. Gallardo R. Steyaert J. Kortholt A.. (2019). Structure and nucleotide-induced conformational dynamics of the Chlorobium tepidum Roco protein. Biochem. J. 476, 51–66. doi: 10.1042/BCJ20180803 30538153

[B14] DeYoung B. J. Innes R. W. (2006). Plant NBS-LRR proteins in pathogen sensing and host defense. Nat. Immunol. 7, 1243–1249. doi: 10.1038/ni1410 17110940 PMC1973153

[B15] Dittami S. M. Scornet D. Petit J. L. Ségurens B. Silva C. D. Corre E.. (2009). Global expression analysis of the brown alga Ectocarpus siliculosus (Phaeophyceae) reveals large-scale reprogramming of the transcriptome in response to abiotic stress. Genome Biol. 10, R66–R66. doi: 10.1186/gb-2009-10-6-r66 19531237 PMC2718500

[B16] Dorrell R. G. Gile G. Mccallum G. Méheust R. Bapteste E. P. Klinger C. M.. (2017). Chimeric origins of ochrophytes and haptophytes revealed through an ancient plastid proteome. eLife 6, e23717. doi: 10.7554/eLife.23717.055 28498102 PMC5462543

[B17] Edgar R. C. (2021). MUSCLE v5 enables improved estimates of phylogenetic tree confidence by ensemble bootstrapping. bioRxiv. doi: 10.1101/2021.06.20.449169

[B18] Gardet A. Benita Y. Li C. Sands B. E. Ballester I. Stevens C.. (2010). LRRK2 is involved in the IFN-γ response and host response to pathogens. J. Immunol. 185, 5577–5585. doi: 10.4049/jimmunol.1000548 20921534 PMC3156100

[B19] Hakimi M. Selvanantham T. Swinton E. Padmore R. F. Tong Y. Kabbach G.. (2011). Parkinson’s disease-linked LRRK2 is expressed in circulating and tissue immune cells and upregulated following recognition of microbial structures. J. Neural Transm. 118, 795–808. doi: 10.1007/s00702-011-0653-2 21552986 PMC3376651

[B20] Inbal B. Cohen O. Polak-Charcon S. Kopolovic J. Vadai E. Eisenbach L.. (1997). DAP kinase links the control of apoptosis to metastasis. Nature 390, 180–184. doi: 10.1038/36599 9367156

[B21] Jordan T. Schornack S. Lahaye T. (2002). Alternative splicing of transcripts encoding Toll-like plant resistance proteins–what's the functional relevance to innate immunity? Trends Plant Sci. 7, 392–398. doi: 10.1016/S1360-1385(02)02311-7 12234730

[B22] Keeling P. J. (2010). The endosymbiotic origin, diversification and fate of plastids. Philos. Trans. R Soc. B: Biol. Sci. 365, 729–748. doi: 10.1098/rstb.2009.0103 PMC2817223 20124341

[B23] Kobe B. Deisenhofer J. (1994). The leucine-rich repeat: a versatile binding motif. Trends Biochem. Sci. 19, 415–421. doi: 10.1016/0968-0004(94)90090-6 7817399

[B24] Kobe B. Kajava A. V. (2001). The leucine-rich repeat as a protein recognition motif. Curr. Opin. Struct. Biol. 11, 725–732. doi: 10.1016/S0959-440X(01)00266-4 11751054

[B25] Koo Y. B. JI I. Slaughter R. G. JI T. H. (1991). Structure of the luteinizing hormone receptor gene and multiple exons of the coding sequence. Endocrinology 128, 2297–2308. doi: 10.1210/endo-128-5-2297 2019252

[B26] Kortholt A. van Egmond W. N. Plak K. Bosgraaf L. Keizer-Gunnink I. van Haastert P. J. (2012). Multiple regulatory mechanisms for the Dictyostelium Roco protein GbpC. J. Biol. Chem. 287, 2749–2758. doi: 10.1074/jbc.M111.315739 22119747 PMC3268432

[B27] Kozlov A. M. Darriba D. Flouri T. Morel B. Stamatakis A. (2019). RAxML-NG: a fast, scalable and user-friendly tool for maximum likelihood phylogenetic inference. Bioinformatics 35, 4453–4455. doi: 10.1093/bioinformatics/btz305 31070718 PMC6821337

[B28] Lewis P. A. (2009). The function of ROCO proteins in health and disease. Biol. Cell 101, 183–191. doi: 10.1042/BC20080053 19152505

[B29] Lipinska A. P. Serrano-Serrano M. L. Cormier A. Peters A. F. Kogame K. Cock J. M.. (2019). Rapid turnover of life-cycle-related genes in the brown algae. Genome Biol. 20, 1–18. doi: 10.1101/290809 30764885 PMC6374913

[B30] Marín I. (2006). The Parkinson disease gene LRRK2: evolutionary and structural insights. Mol. Biol. Evol. 23, 2423–2433. doi: 10.1093/molbev/msl114 16966681

[B31] Marín I. (2008). Ancient origin of the Parkinson disease gene LRRK2. J. Mol. Evol. 67, 41–50. doi: 10.1007/s00239-008-9122-4 18523712

[B32] Marín I. van Egmond W. N. van Haastert P. J. (2008). The Roco protein family: a functional perspective. FASEB J. 22, 3103–3110. doi: 10.1096/fj.08-111310 18523161

[B33] Mastrangelo A. M. Marone D. Laidò G. De Leonardis A. M. De Vita P. (2012). Alternative splicing: enhancing ability to cope with stress via transcriptome plasticity. Plant Sci. 185, 40–49. doi: 10.1016/j.plantsci.2011.09.006 22325865

[B34] Meyers B. C. Kaushik S. Nandety R. S. (2005). Evolving disease resistance genes. Curr. Opin. Plant Biol. 8, 129–134. doi: 10.1016/j.pbi.2005.01.002 15752991

[B35] Mistry J. Finn R. D. Eddy S. R. Bateman A. Punta M. (2013). Challenges in homology search: HMMER3 and convergent evolution of coiled-coil regions. Nucleic Acids Res. 41, e121–e121. doi: 10.1093/nar/gkt263 23598997 PMC3695513

[B36] Ng A. C. Eisenberg J. M. Heath R. J. Huett A. Robinson C. M. Nau G. J.. (2011). Human leucine-rich repeat proteins: a genome-wide bioinformatic categorization and functional analysis in innate immunity. Proc. Natl. Acad. Sci. 108, 4631–4638. doi: 10.1073/pnas.1000093107 20616063 PMC3063585

[B37] Nishitsuji K. Arimoto A. Higa Y. Mekaru M. Kawamitsu M. Satoh N.. (2019). Draft genome of the brown alga, Nemacystus decipiens, Onna-1 strain: fusion of genes involved in the sulfated fucan biosynthesis pathway. Sci. Rep. 9, 1–11. doi: 10.1038/s41598-019-40955-2 30872679 PMC6418280

[B38] Nishitsuji K. Arimoto A. Iwai K. Sudo Y. Hisata K. Fujie M.. (2016). A draft genome of the brown alga, Cladosiphon okamuranus, S-strain: a platform for future studies of ‘mozuku’biology. DNA Res. 23, 561–570. doi: 10.1093/dnares/dsw039 27501718 PMC5144679

[B39] Ritter A. Dittami S. M. Goulitquer S. Correa J. A. Boyen C. Potin P.. (2014). Transcriptomic and metabolomic analysis of copper stress acclimation in Ectocarpus siliculosus highlights signaling and tolerance mechanisms in brown algae. BMC Plant Biol. 14, 116. doi: 10.1186/1471-2229-14-116 24885189 PMC4108028

[B40] Schmucker D. Chen B. (2009). Dscam and DSCAM: complex genes in simple animals, complex animals yet simple genes. Genes Dev. 23, 147–156. doi: 10.1101/gad.1752909 19171779

[B41] Smith P. H. Mwangi J. M. Afrane Y. A. Yan G. Obbard D. J. Ranford-Cartwright L. C.. (2011). Alternative splicing of the Anopheles Gambiae Dscam gene in diverse Plasmodium falciparum infections. Malaria J. 10, 1–7. doi: 10.1186/1475-2875-10-156 PMC3118162 21651790

[B42] Teng L. Liang M. Wang C. Li Y. Urbach J. M. Kobe B.. (2023). Exon shuffling potentiates a diverse repertoire of brown algal NB-ARC-TPR candidate immune receptor proteins via alternative splicing. Plant J. 114, 246–261. doi: 10.1111/tpj.16131 36738111

[B43] Terheyden S. (2018). Structural and biochemical characterization of Roco proteins. University of Groningen, Groningen.

[B44] Thomas F. Cosse A. Le Panse S. Kloareg B. Potin P. Leblanc C. (2014). Kelps feature systemic defense responses: insights into the evolution of innate immunity in multicellular eukaryotes. New Phytol. 204, 567–576. doi: 10.1111/nph.12925 25041157

[B45] van Egmond W. N. Kortholt A. Plak K. Bosgraaf L. Bosgraaf S. Keizer-Gunnink I.. (2008). Intramolecular activation mechanism of the Dictyostelium LRRK2 homolog Roco protein GbpC. J. Biol. Chem. 283, 30412–30420. doi: 10.1074/jbc.M804265200 18703517 PMC2662088

[B46] Wauters L. Versées W. Kortholt A. (2019). Roco proteins: GTPases with a baroque structure and mechanism. Int. J. Mol. Sci. 20, 147. doi: 10.3390/ijms20010147 30609797 PMC6337361

[B47] Yang X. Coulombe-Huntington J. Kang S. Sheynkman G. M. Hao T. Richardson A.. (2016). Widespread expansion of protein interaction capabilities by alternative splicing. Cell 164, 805–817. doi: 10.1016/j.cell.2016.01.029 26871637 PMC4882190

[B48] Zambounis A. Elias M. Sterck L. Maumus F. Gachon C. M. M. (2012). Highly dynamic exon shuffling in candidate pathogen receptors … what if brown algae were capable of adaptive immunity? Mol. Biol. Evol. 29, 1263–1276. doi: 10.1093/molbev/msr296 22144640 PMC3341825

[B49] Zhang X. Fan X. Wang Y. Xu D. Zhang J. Ye N. (2021a). Exploring core response mechanisms to multiple environmental stressors via A genome-wide study in the brown alga saccharina japonica (Laminariales, phaeophyceae). J. Phycology 57, 345–354. doi: 10.1111/jpy.13108 33211355

[B50] Zhang X. Zhang J. Wang Y. Xu D. Fan X. Zhang Y.. (2021b). The oxylipin messenger 1-octen-3-ol induced rapid responses in kelp Macrocystis pyrifera. Physiologia Plantarum 172, 1641–1652. doi: 10.1111/ppl.13358 33547806

