# Supplementary material for: Exon shuffling and alternative splicing of ROCO genes in brown algae enables a diverse repertoire of candidate immune receptors
Source: Front Plant Sci. 2024 Aug 23;15:1445022. doi: 10.3389/fpls.2024.1445022 (PMC11378527; doi:10.3389/fpls.2024.1445022)
Supplement: Supplementary file 1 [file DataSheet1.docx]

Exon shuffling and alternative splicing of ROCO genes in brown algae enables a diverse repertoire of candidate immune receptors

Table S1. The sequence information of the brown algal ROCO genes.

*Ectocarpus* sp.

| Sequence ID | MW（kDa） | pI | TH | Length(aa) |  | | SP | Subcellular localization | |
| --- | --- | --- | --- | --- | --- | --- | --- | --- | --- |
| Ec-01_001800 | 143.36 | 5.65 | 0 | 1316 |  | - | | | Cp |
| Ec-01_001850 | 92.29 | 6.09 | 1 | 844 |  | - | | | Cp |
| Ec-01_002500 | 120.71 | 5.35 | 0 | 1114 |  | - | | | Cp |
| Ec-02_003690 | 117.53 | 6.15 | 2 | 1074 |  | - | | | Cp |
| Ec-03_000130 | 116.93 | 6.88 | 0 | 1056 |  | - | | | Cp |
| Ec-03_000700 | 118.94 | 5.75 | 0 | 1093 |  | - | | | Nu |
| Ec-03_000710 | 136.58 | 5.43 | 0 | 1236 |  | - | | | Chl、Cp、Nu |
| Ec-03_000720 | 124.56 | 5.31 | 0 | 1136 |  | - | | | Cp |
| Ec-03_000770 | 109.39 | 5.63 | 0 | 987 |  | - | | | Cp |
| Ec-03_000780 | 136.61 | 5.77 | 0 | 1245 |  | - | | | Cp |
| Ec-03_001790 | 165.51 | 5.68 | 0 | 1527 |  | - | | | Cp |
| Ec-06_001640 | 143.29 | 5.69 | 0 | 1303 |  | - | | | Cp、Nu |
| Ec-08_002960 | 236.65 | 5.02 | 0 | 2129 |  | - | | | Cp |
| Ec-10_000570 | 119.01 | 5.71 | 0 | 1091 |  | - | | | Cp |
| Ec-10_000810 | 101.42 | 5.93 | 1 | 928 |  | - | | | Cp |
| Ec-10_004370 | 117.56 | 6.57 | 1 | 1078 |  | - | | | Cp |
| Ec-10_004500 | 137.20 | 5.59 | 1 | 1261 |  | - | | | Cp |
| Ec-11_005360 | 129.44 | 6.97 | 0 | 1166 |  | - | | | Cp |
| Ec-12_003110 | 143.50 | 5.34 | 0 | 1312 |  | - | | | Cp、Nu |
| Ec-15_004730 | 134.92 | 5.41 | 0 | 1243 |  | - | | | Cp |
| Ec-15_004840 | 139.34 | 5.39 | 0 | 1282 |  | - | | | Cp |
| Ec-16_004800 | 125.22 | 6.56 | 0 | 1148 |  | - | | | Cp |
| Ec-16_004940 | 237.92 | 6.38 | 0 | 2275 |  | - | | | Cp |
| Ec-19_000270 | 126.14 | 5.67 | 0 | 1158 |  | - | | | Cp |
| Ec-19_000290 | 119.66 | 5.88 | 0 | 1091 |  | - | | | Cp |
| Ec-21_005740 | 128.11 | 5.59 | 0 | 1159 |  | - | | | Cp |
| Ec-23_004280 | 87.96 | 5.40 | 0 | 800 |  | - | | | Cp、Nu |
| Ec-25_000790 | 136.67 | 6.37 | 0 | 1246 |  | - | | | Cp |
| Ec-25_003370 | 242.81 | 9.09 | 0 | 2294 |  | - | | | Cp、Nu |
| Ec-27_002510 | 119.04 | 5.47 | 0 | 1073 |  | - | | | Cp |
| Ec-28_001230 | 96.43 | 5.55 | 0 | 882 |  | - | | | Cp |
| Average length |  |  |  | 1243 |  |  | | |  |

*Saccharina japonica*

| Gene ID | Length(AA) | MW(kDa) | TH | PI | SP | Subcellular |
| --- | --- | --- | --- | --- | --- | --- |
| SJ02233 | 1314 | 142.84 | 2 | 5.51 | - | Cp |
| SJ03714 | 1312 | 142244.68 | 0 | 5.77 | - | Cp、Er、Mt、Nu |
| SJ05253 | 1269 | 140211.24 | 0 | 6.93 | - | Cp |
| SJ08268 | 1050 | 115942.34 | 0 | 6.20 | - | Cp |
| SJ08269 | 978 | 107.68 | 0 | 5.7 | - | Cp |
| SJ08396 | 1145 | 128.66 | 0 | 5.63 | - | Cp |
| SJ10859 | 1110 | 121026.65 | 0 | 6.10 | - | Cp |
| SJ12675 | 1253 | 135365.13 | 1 | 6.01 | - | Cp |
| SJ13174 | 1047 | 116470.06 | 0 | 5.87 | - | Cp |
| SJ16113 | 2245 | 232479.10 | 0 | 5.94 | - | Cp |
| SJ20422 | 1041 | 115702.41 | 0 | 6.15 | - | Cp |
| Average length | 1251 |  |  |  |  |  |

*Cladosiphon okamuranus*

| sequence | MW(Da) | PI | Length (aa) | TH | SP | Subcellular localization |
| --- | --- | --- | --- | --- | --- | --- |
| g4349.t1 | 115.85 | 6.13 | 1043 | 2 | - | Cp |
| g6184.t1 | 124.7 | 7.91 | 1117 | 0 | - | Cp |
| g6853.t1 | 97.9 | 5.95 | 895 | 2 | - | Cm, Cp |
| g7130.t1 | 198.2 | 6.37 | 1793 | 11 | - | Cm, Cp, Nu |
| g8089.t1 | 173.5 | 5.60 | 1587 | 0 | - | Cp, Nu |
| g8092.t1 | 135.13 | 5.64 | 1228 | 0 | - | Cp |
| g8941.t1 | 121.6 | 5.68 | 1106 | 0 | - | Cp |
| g9797.t1 | 126.02 | 5.98 | 1140 | 0 | - | Chl, Cp, Nu |
| g10043.t1 | 43.27 | 9.39 | 386 | 0 | - | Cm, Cp, Mt, Nu |
| g10191.t1 | 101.36 | 6.05 | 929 | 0 | - | Cp, Mt, Nu |
| g11199.t1 | 159.6 | 5.79 | 1449 | 0 | - | Cp, Nu |
| g12476.t1 | 104.19 | 6.21 | 944 | 0 | - | Cp |
| g12713.t1 | 249.59 | 8.63 | 2376 | 0 | - | Nu |
| g13758.t1 | 147.14 | 5.58 | 1339 | 0 | - | Cp |
| g14342.t1 | 214.07 | 5.90 | 1906 | 0 | - | Cp |
| g15968.t1 | 65.2 | 5.10 | 597 | 0 | - | Cp, Nu |
| g16363.t1 | 123.57 | 5.98 | 1120 | 0 | - | Cp |
| g17016.t1 | 46.36 | 5.49 | 460 | 0 | - | Er, Nu |
| g17155.t1 | 160.45 | 6.42 | 1453 | 8 | - | Cp |
| Average length |  |  | 1203 |  |  |  |

*Nemacystus decipiens*

| **Gene ID** | **Length(aa)** | **MW(kDa)** | **TH** | **PI** | **SP** | **Subcellular localization** |
| --- | --- | --- | --- | --- | --- | --- |
| g1247.t1 | 1154 | 126.67 | 0 | 5.88 | - | Nu |
| g1285.t1 | 2368 | 256.26 | 0 | 6.60 | - | Cp、Nu |
| g1813.t1 | 1082 | 117.52 | 0 | 5.68 | - | Cp |
| g2976.t1 | 1178 | 127.58 | 0 | 5.98 | - | Cp |
| g3577.t1 | 762 | 83.54 | 0 | 6.43 | - | Cp |
| g3590.t1 | 999 | 108.95 | 3 | 5.66 | - | Cp |
| g3608.t1 | 1163 | 128.08 | 0 | 5.52 | - | Cp |
| g4975.t1 | 1258 | 138.90 | 0 | 5.97 | - | Cp |
| g5738.t1 | 967 | 106.01 | 2 | 5.76 | - | Cp |
| g5808.t1 | 2127 | 230.23 | 0 | 5.87 | - | Cp、Nu、Mt |
| g5836.t1 | 1924 | 206.45 | 0 | 5.79 | - | Cp、Nu |
| g6262.t1 | 1251 | 136.71 | 0 | 5.68 | - | CM、Cp |
| g7447.t1 | 935 | 101.73 | 0 | 5.30 | - | Cp |
| g7449.t1 | 1187 | 130.09 | 0 | 5.24 | - | Cp |
| g7583.t1 | 1151 | 125.70 | 2 | 5.52 | - | CM、Cp |
| g7876.t1 | 1501 | 165.00 | 0 | 6.31 | - | Cp |
| g8047.t1 | 1225 | 133.77 | 1 | 7.52 | - | CM、Cp |
| g8079.t1 | 2408 | 252.82 | 0 | 8.92 | - | Cp、Nu |
| g8726.t1 | 2283 | 243.48 | 2 | 6.18 | - | Cp、Nu |
| g8764.t1 | 2356 | 244.79 | 0 | 6.09 | - | Cp |
| g9276.t1 | 1393 | 152.79 | 0 | 5.30 | - | Cp |
| g9538.t1 | 1393 | 135.35 | 3 | 5.09 | - | Chl、Cp、Nu |
| g10183.t1 | 1521 | 167.36 | 0 | 5.88 | - | Cp |
| g10379.t1 | 1000 | 110.27 | 1 | 5.32 | - | Cp、Nu |
| g10841.t1 | 1191 | 129.92 | 0 | 5.40 | - | Cp |
| g11041.t1 | 1482 | 163.49 | 0 | 5.16 | - | Cp、Nu |
| g11302.t1 | 475 | 51.12 | 0 | 5.53 | - | Er、Nu |
| g11487.t1 | 1124 | 123.30 | 0 | 5.68 | - | CM、Cp、Chl |
| g11847.t1 | 1211 | 133.63 | 3 | 6.02 | - | CM、Cp、Nu |
| g12085.t1 | 2213 | 242.01 | 0 | 5.63 | - | Cp |
| g12771.t1 | 950 | 102.51 | 2 | 5.91 | - | Cp、Nu |
| g12804.t1 | 975 | 105.83 | 1 | 5.88 | - | Cp、Nu |
| g13180.t1 | 739 | 80.85 | 0 | 5.58 | - | Cp |
| g14316.t1 | 1008 | 109.99 | 2 | 5.90 | - | CM、Cp |
| g14419.t1 | 985 | 108.26 | 1 | 5.53 | - | Cp |
| g15068.t1 | 820 | 89.91 | 0 | 5.49 | - | CM、Cp、Nu |
| g15092.t1 | 1311 | 144.55 | 0 | 6.34 | - | Cp、Nu |
| g15166.t1 | 1451 | 161.52 | 0 | 6.15 | - | Cp |
| g15205.t1 | 495 | 53.82 | 0 | 5.21 | - | Cp |
| g15229.t1 | 773 | 84.68 | 0 | 5.34 | - | Cp |
| g15262.t1 | 1237 | 135.79 | 0 | 5.10 | - | Chl、Cp、Nu |
| g15339.t1 | 300 | 32.45 | 0 | 9.26 | - | CM、Cp、Er |
| g15364.t1 | 407 | 44.21 | 0 | 8.24 | - | Cp |
| g15937.t1 | 1064 | 117.33 | 2 | 5.33 | - | Cp |
| g15979.t1 | 1274 | 138.52 | 0 | 5.50 | - | Cp |
| g15981.t1 | 1696 | 185.75 | 0 | 5.68 | - | Chl、Nu、Cp |
| g16322.t1 | 1887 | 206.68 | 0 | 6.21 | - | Cp、Nu |
| Average length | 1269 |  |  |  |  |  |

note： Nu, Nuclear; Cp, Cytoplasm、 Er, Endoplasmic reticulum、 Chl, Chloroplast, CM, Cell membrane

Table S2. The representative ROC domain sequences of brown algae used in online BLASTP.

| **Gene ID.** | **ROC domain** |
| --- | --- |
| Cok_S_s150_12476.t1 | GRTSSNRLKVVLVGLGNAGKTSVAVRLEGPASSKPLPTAEERTVGVEIRDIQLGSGPVNGGSRGDAELNVKLWDFAGQRAYYDTHQMFLTPGALFVLVVDIFAYSEDGHSREDALEQWLDILQARVPGSVVLLVGTHSDLFDSPADCSKRVKLFLKDKEDIMDRIDRECKSARDRARKESAENSDWEYAGVQDSRRYQPLRIVVVSELLALNLTPSGGITTAQLKEHLRELPTAF |
| Cok_S_s354_17155.t1 | GVQKSWMIKVVLVGAVCAGKSSVVASLTARQPRQVPLAQRTRGVDVHVQKPFRPGGSMAKLVFWDFAGHDDYYSTHSLFLSDGALLLLVVDLARFVDDPASRSNAVHIWLDALLCRTPGAVVQIIATHTDELGGEHDVETVVNELRQVVAAHLKAKCGEYERGWKSGGRTEEMPAPPTLRVVYQIHTVSCKTGANWPELGRAIGDLAGHG |
| Ec-21_005740 | GRAWCNRLKVVLVGLGEAGKTSIATRLEDRLASSCPKPEERTVGVEIRDMKLGPGPTNEGSGPNVELDVSLWDFAGQRAYYDTHQMFLTPGALFVLVVDMFTYAEGHSREDALEQWLDILQARVPGSVVLLVGTHTDLFEDNAAECTERTDSFKKDVEDVMTRMRCQCDSAKDRTETELGKGHPDLEGNPRYQPLRVVLEEDLLALDLTSSDGQDIDLLQHRLEHLAYNG |
| Ec-10_000570 | AVTRPLKVVIVGKETVGKTSLRRSIKTGKPCMTRGGGEESTVHVDVEDHDLDGHPIRMFDCAGQVVYYGLLQLFLTPRAVYLLVWDAAKASEMEGLNLEDLAIAPWLRHLTFRVPDASVILVGNKCDRVVRTRRTAVAVDVEHESRQWLESWIEIARGHQ |
| g7876.t1 | GEGKSNRLKMVLVGLAEAGKTTVVRHFTGGPVPRRPDRTVGIEITKDWRPSLEVPLQVSIWDFAGQADYYSSHQLFLTKGALCLLVVDLHAFSKEVGSDEDNFTDPHGRIYWWLEMIHIRVPGAAIALVGTHVDDMEKDGVDSERAGIRLYTAVSKFIESKVMNASNGNSDRSIDTESTSKTDQLRDRVQAFPPGV |
| g7449.t1 | PRTMIKVVLVGQEGAGKTSLRQSIRNRRPTPTGGPAESTVQIDVEEVKMDVDGVSLRVYDCAGQVAYTGLLQMFLSPRAVSLLVCNAEAFGQGDDCTSDKVQLTQDLSKLRELRVCDWLRSLSFRIPDSDVVVVATKCDLVAAGTAASLAGKMERAIRRWLEYWRGSDMTAVRVEDGVS |
| SJ20422 | TSRNSWTLKVVVVGAVCAGKSSVVRSLMAREALLVPFADRTRGVDVHVEEPFKPDESRPVELVFWDFAGHDDYYSTHSLFISEGALFLLVVDVARFVEDSSSRAGAIHIWLDTLLCRTPGAVVQIVATHIDQLGGSLEDAVQQLRQVVSDHFAAKRDEHERGWVKSGQEKGMHALPTLRVVDEIIAVSCKEGTNLPALGEALGNLAADG |

**Table S3.** The parameters of selection analysis using site model on LRR sequences of *S. japonica*.

| **Model=0: one-ratio** | | **ω: 0.3622** |  |  |  |
| --- | --- | --- | --- | --- | --- |
| Model=1: NearlyNeutral LnL: -3496.622 | | |  |  |  |
|  | p: 0.47826 0.52174 | |  |  |  |
|  | ω: 0.09079 1.00000 | |  |  |  |
| Model 2: Positive Selection LnL: -3496.045 | | |  |  |  |
| p: 0.47826 0.41574 0.10600  ω: 0.09485 1.00000 1.43656 | | | |  |  |
| Positively selected sites: 14 T, 16N, 19G | | | |  |  |
| Model 7: beta | | LnL: -3493.539 |  |  |  |
| p = 0.46431 q = 0.55114 | | | |  |  |
| p: 0.10000 0.10000 0.10000 0.10000 0.10000 0.10000 0.10000 0.10000 0.10000 0.10000 | | | |  |  |
| ω: 0.00346 0.03650 0.10719 0.21324 0.34777 0.50008 0.65648 0.80127 0.91772 0.98850 | | | |  |  |
| Model 8: beta&ω>1 LnL: -3492.5706 | | | |  |  |
| p0 = 0.80011 p = 0.82737 q = 1.63189 | | | |  |  |
| (p1 = 0.19989) ω = 1.05402 | | | |  | |
| Positively selected sites: 14T, 16N, 18S, 19G | | | |  |  |

**
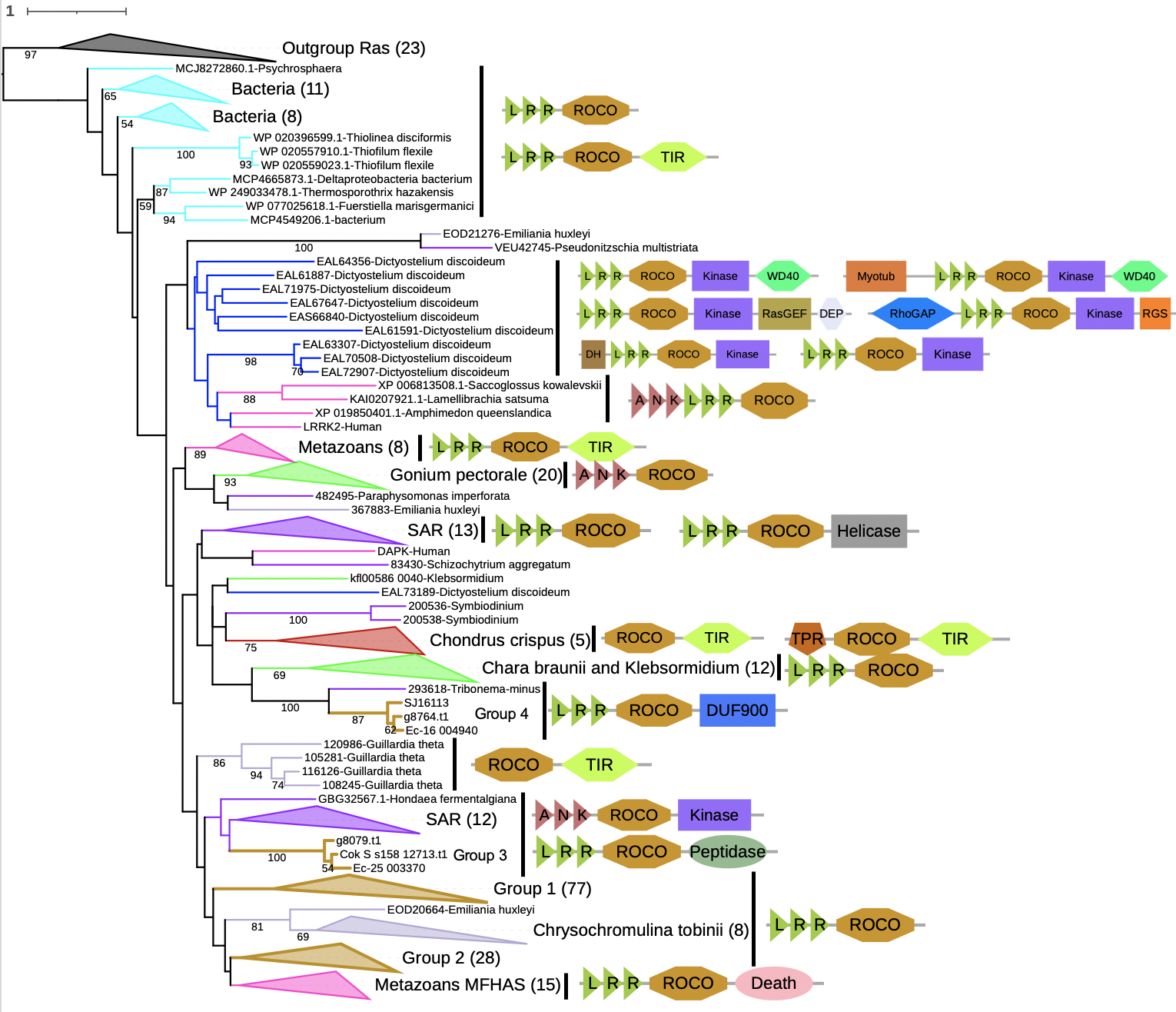
**

**Figure S1.** A total of 267 ROC domain sequences and 17 Ras sequences were aligned using MUSCLE5. The maximum-likelihood (ML) tree was generated using RAxML-NG with the WAG+G4 model predicted by ModelTest-NG. Domain combinations in each clade are shown on the right.


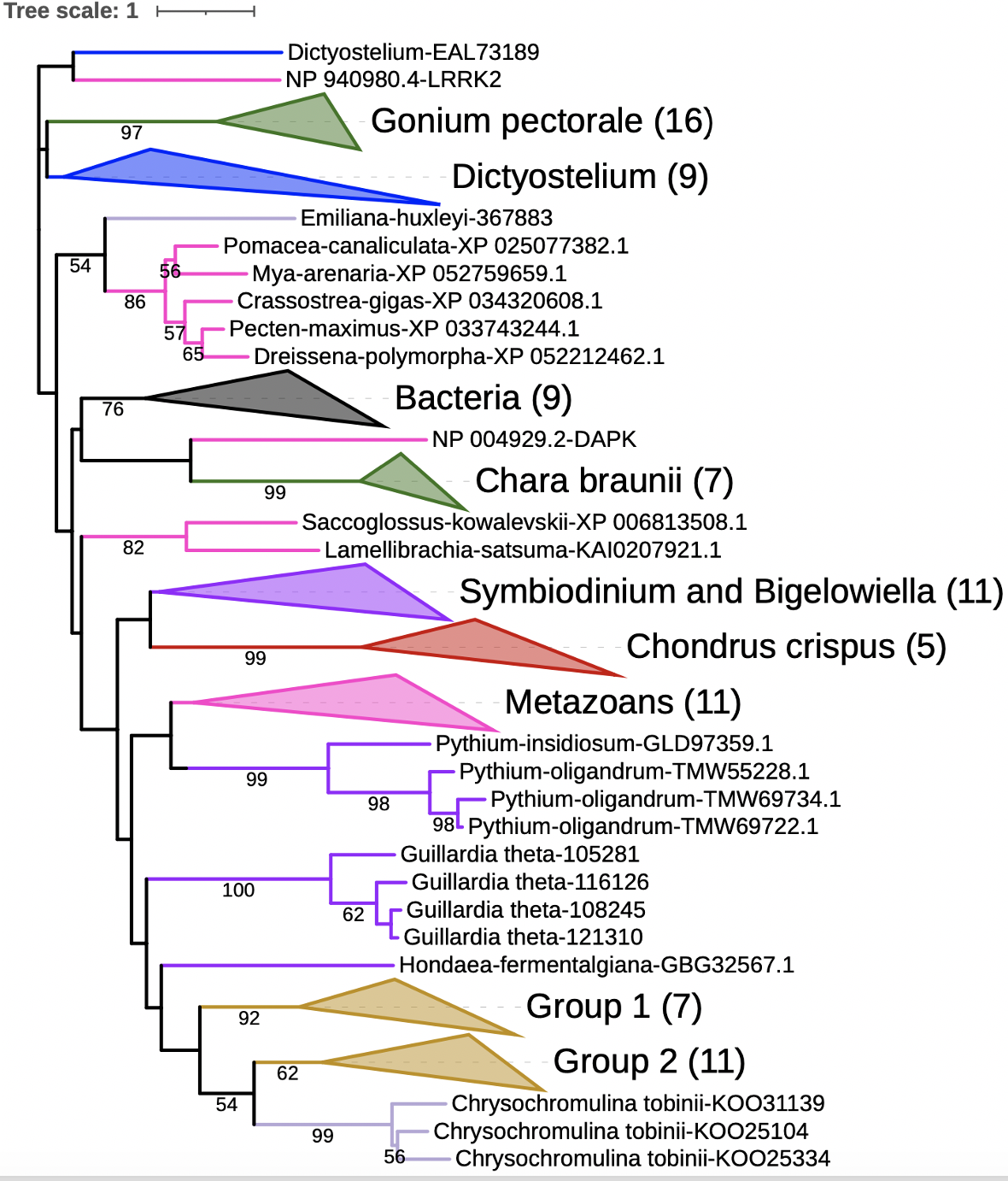


**Figure S2**. Phylogenetic tree of the 111 COR domain sequences found in the ROCO sequences in figure 2A. ML tree was generated using RAxML-NG with the LG+I+G4 model predicted by ModelTest-NG. Branch color represents different kingdoms of life

**
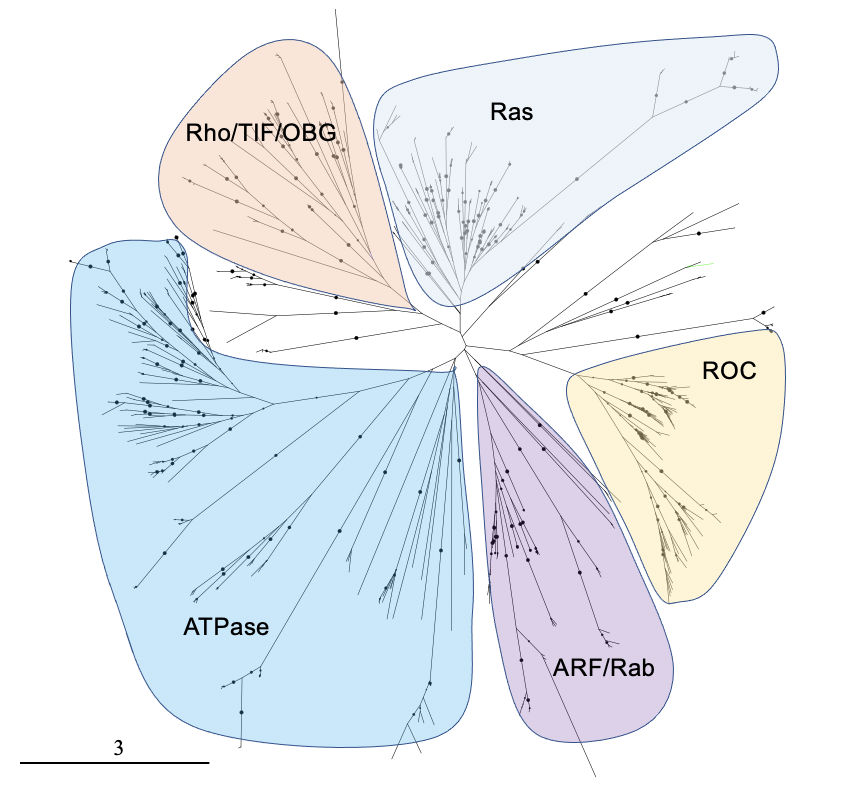
**

**Figure S3.** Phylogenetic tree of 491 brown algal ROC domain-containing genes and other small GTPase superfamily genes. the sequences were obtained by using brown algal ROC domain as query to perform hmmsearch in four brown algal proteomes.
